# Will Protective Innovations Like the “Airway Box” Become Routine Practice After the Pandemic?: An Opinion Survey

**DOI:** 10.7759/cureus.13258

**Published:** 2021-02-10

**Authors:** Pavithra Ellison, Benton Nanners, Gregory Schaefer, Austin Krueger, Vipul Dhumak, Jason L Shepherd, Khoa Nguyen, Kathrin Allen, Matthew Ellison

**Affiliations:** 1 Anesthesiology, West Virginia University School of Medicine, Morgantown, USA; 2 Surgery, West Virginia University School of Medicine, Morgantown, USA; 3 Anesthesiology and Critical Care, West Virginia University School of Medicine, Morgantown, USA

**Keywords:** intubation, intubation box, personal protective equipment, medical device

## Abstract

Background

Tracheal intubation carries an elevated risk of exposure to severe acute respiratory syndrome coronavirus 2 (SARS-CoV-2) due to the generation of aerosols containing high concentrations of the virus. An airway box was designed to mitigate the exposure of healthcare professionals performing intubations.

Aim

We evaluated usability and sustainability in the routine practice of the "airway box" as a protective device during high-risk airway procedures.

Materials and methods

After institutional review board approval, clinicians were educated on using the device through simulation, intranet learning modules, and emailed resources. The airway box was made available in the emergency department, critical care units, perioperative area, and operating rooms. QR codes affixed to the box, emailed, and displayed in common areas provided easy access to complete a REDcap survey (Vanderbilt University Nashville, USA) eliciting providers’ experience. Data was collected and analyzed between April 1 and July 31, 2020, on REDcap, and the results were analyzed.

Results

687 emergent intubations took place. 232 were performed by anesthesiologists, 315 by emergency department providers, and 140 by critical care specialists. 39 surveys were completed, 29 from intubations in the operating room, three from the critical care units, five from interventional radiology suites, and two perioperatively. Providers found the device to be readily available, with a score of 4.51/5, and the majority of providers, 60%, found the device easy to use, rating it either a 4 or 5 out of 5. Providers acquired a mean Mallampati score of 1.75 and 1.40 mean laryngoscopic grade view.

Conclusion

Intubation boxes may effectively mitigate high-risk viral exposure during airway procedures. Survey responses show that devices were easy to use and did not significantly affect visualization of the airway. Similar to mask use, enclosure devices in clinical practice could become a vital part of medical protective equipment even after the SARS-CoV-2 pandemic if they are effectively implemented.

## Introduction

Severe acute respiratory syndrome coronavirus 2 (SARS-CoV-2) has led to over 105 million cases worldwide with over 2.1 million deaths, including 26.9 million cases and over 460,000 deaths in the United States as of February 2021. Studies have found that tracheal intubation has an increased risk of exposure to certain pathogens like SARS-CoV-2 [1, 2]. Increased risk of transmission has changed our daily routines and healthcare practices. An airway box was designed by Dr. Hsien-Yung Lai to help decrease exposure as it is a physical barrier between the provider and patient to decrease the risk of a direct spray of aerosolized viral particles during the manipulation of the airway [3]. The study showed that without any barrier implementation, the particles spread to a two-meter radius. With the airway box, the particles were contained within the device [4]. Healthcare providers are essential in the fight against SARS-CoV-2 and protecting clinicians is a priority. Large scale personal protective equipment (PPE) shortages have created the necessity to conserve resources while limiting exposures that spread SARS-CoV-2. Some procedures confer a higher risk of viral aerosolization and exposure, therefore, making them high-priority intervention targets to limit the spread.

Intubation and extubation of the airway are examples of high-risk procedures that endanger the provider due to potential direct aerosolization of viral particles to the provider's face. This direct spray of aerosolized droplet particles larger than 5µm presents a plausibly significant exposure risk for SARS-CoV-2 [5]. Up to 8% of SARS-CoV-2 cases require intubation, and data from the World Health Organization (WHO) suggests that 80% of SARS-CoV-2 infections are asymptomatic or present mild symptoms [6]. When SARS-CoV-2 patients and asymptomatic carriers require intubations, it creates a substantial risk to the provider. Many facilities require a SARS-CoV-2 test for patients prior to entering the hospital for scheduled procedures. Even with testing, there is a risk of intubating patients with undetected infections. An article in the *New England Journal of Medicine* found SARS-CoV2 tests have a 2-29% false-negative rate [7]. If the airway box is utilized during all intubation/extubation events, it may significantly limit the undetected exposure when a provider perceives no risk of SARS-CoV-2 exposure from the patient. The device’s merit relies on the effective low-cost reusable nature of its potential addition to SARS-CoV-2 PPE. 

The vast impact of the pandemic necessitates an evaluation of all options to decrease spread and protect clinicians. The Association of Anesthetists discusses the need for modifications to reduce nosocomial infections citing that more than 20% of SARS-CoV-2 infections are healthcare workers [8]. Utilizing the airway box in clinical practice may decrease the risk of transmission. In theory, ensuring total paralysis during intubation should prevent aerosol formation from respiration, gagging, and coughing but comes with its own risks. Another high-risk situation is extubation, where paralysis is not an option and coughing is common. There is limited information about the amount of aerosolization that occurs during extubation. Simply positioning the airway box over the patient's head before intubation, extubation, and during airway manipulation could mitigate transmission risk.

We aimed to evaluate the perceived efficacy, utility, and potential implications of large-scale implementation of the intubation box as a functional piece of PPE against coronavirus disease (COVID-19) transmission. Our assumption is that the low-cost, easy-to-use device decreases potential exposure through the physical barrier separating the provider and patients. Our tertiary care academic center collaborated with the institution’s College of Engineering to design, produce, and implement an intubation protection device [9].

Our goal in this study is to acquire feedback from providers using intubation boxes as an addition to PPE protecting healthcare providers performing high-risk procedures during the SARS-CoV-2 pandemic. The priority of this study was to acquire input from the highly trained specialists on device feasibility, including the ease of use, procedural safety, and grade of intubation view. This would provide valuable insight into clinicians’ viewpoint and help indicate the likelihood of success with implementation.

## Materials and methods

**Figure 1:**
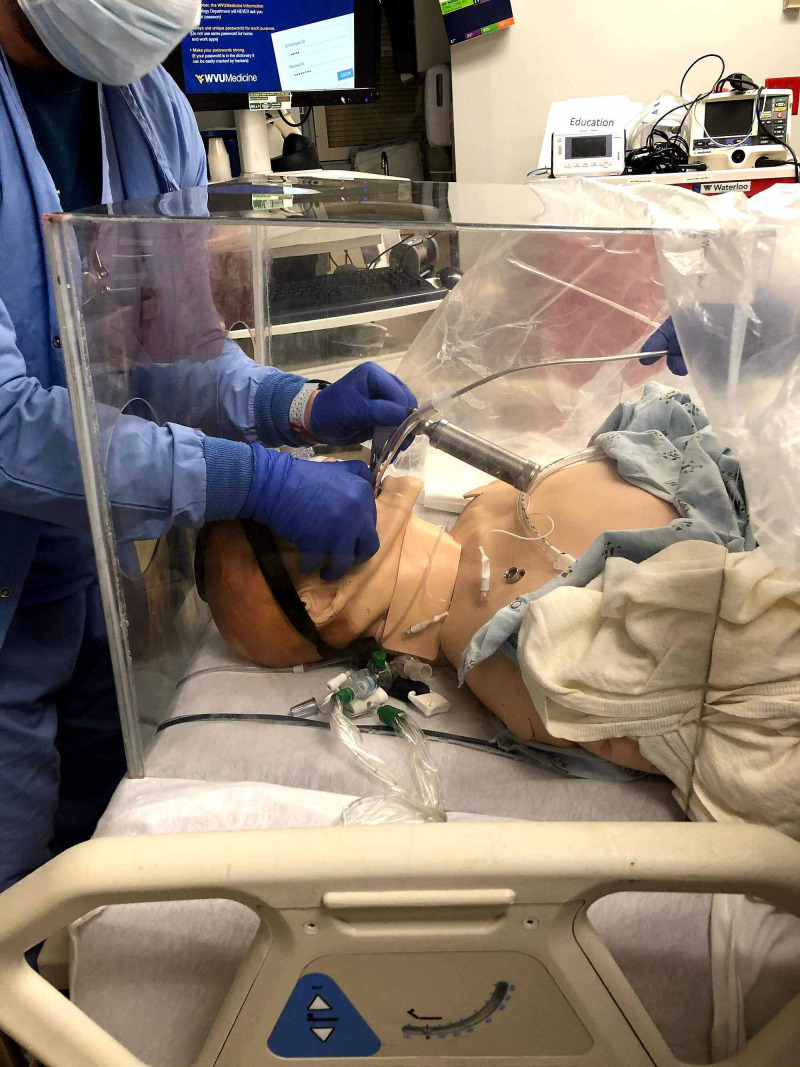
Barrier enclosure device

After the School of Engineering produced the boxes, they were distributed to the operating rooms, intensive care units, emergency department, and floor units. The four-sided 20x20x20-inch barrier enclosure device incorporated two side-holes 6.5 inches in radius for patient access as seen in Figure 1. The clear plexiglass box is placed over the patient’s head during the procedure, the provider gains access to the airway using the holes in the box, and performs the intubation. The box was used in conjunction with plastic drapes placed on the patient contiguous to the box. In conjunction with a whole-body plastic drape, the box is designed to physically block aerosolized particles from spraying into the provider’s face during the procedure.

After Institutional Review Board approval, a REDCap survey was set up to assess interest and feedback on airway boxes. Clinicians were made aware of intubation box availability and were invited to practice intubations in the COVID Intubation Simulation room that was created in our institution. Clinicians also received education via email and intranet learning modules about methods to intubate, extubate, clean, and store the intubation box. Nurse educators, respiratory therapists, and clinicians were all educated about the airway box and were also informed about the study.

Given below is the text introducing the survey and the questionnaire (Table 1).

We request your participation in an Intubation Box utilization research survey being conducted by Dr. Pavithra Ellison, Department of Anesthesiology and Dr. Gregory Schaefer, Division of Trauma and Critical Care at WVU Medicine, to help us better understand utilization of the Intubation Box.

If you agree, you will be asked to answer survey questions on the intubation you participated in. You must be 18 years old and the survey will take less than 3 minutes to complete.

Your response is anonymous, and the data will be kept as confidential as legally possible. All data will be reported in the aggregate. You will not be asked any questions that could lead back to your identity as a participant. Your participation is completely voluntary. You may skip any question that you do not wish to answer, and you may discontinue at any time.

If you have any questions about this research project, please feel free to contact Dr. Ellison or Dr. Schaefer at 304- 598-4000 ext. 4122. If you have any questions about your rights as a research participant, please contact the WVU Office of Human Research Protection by phone at 304-293-7073 or by email at .

**Table 1:** REDCap Survey Questionnaire RSI:  Rapid Sequence Intubation

COVID Intubation Box Survey
Date of Code/Intubation?
What is your clinical role?
Time of Airway Management event?
Age of Patient?
Was the intubation box used for intubation or extubation?
Minutes from RSI meds to successful intubation?
Please rate the availability of bringing the intubation box to bedside?
Please rate ease of use for intubation box?
Please rate impact of intubation box on safety (GREEN = made safer, YELLOW: no change, RED = more dangerous)?
Please indicate any other adjunctive protective methods?
Please indicates devices used for intubation?
How many intubation attempts?
Please indicate Mallampati Score?
Please indicate grade of laryngoscopic view?
Did patient have criteria for difficult airway (HEAVEN, LEMON, etc.)?
What was patient BMI?

Following education of providers, the airway box was made available in the emergency rooms, critical care units, perioperative area, and operating rooms. All staff were encouraged to use it for intubation, extubation, and procedures that manipulated the airway. A QR code to fill out the survey was adhered to the box, emailed to all staff, and posted in common areas. We closed the survey July 31, 2020 and analyzed the results from REDCap. The information was collected using Likert scale for the questions that assess the opinion of the device. Other pertinent data was collected from listed options. Standard statistical analysis of the data was conducted to determine mean, median, standard deviation, and variance.

## Results

Between April 1st and July 31st, 2020, 7,839 general anesthetics occurred, 687 emergent intubations. Of which, 232 were performed by anesthesiologists, 315 in the emergency department, and the remainder were performed by critical care specialists. During the same period, 39 surveys were completed.

Since this is a survey eliciting provider feedback on the intubation box itself, there was no need to review patient charts.

**Figure 2:**
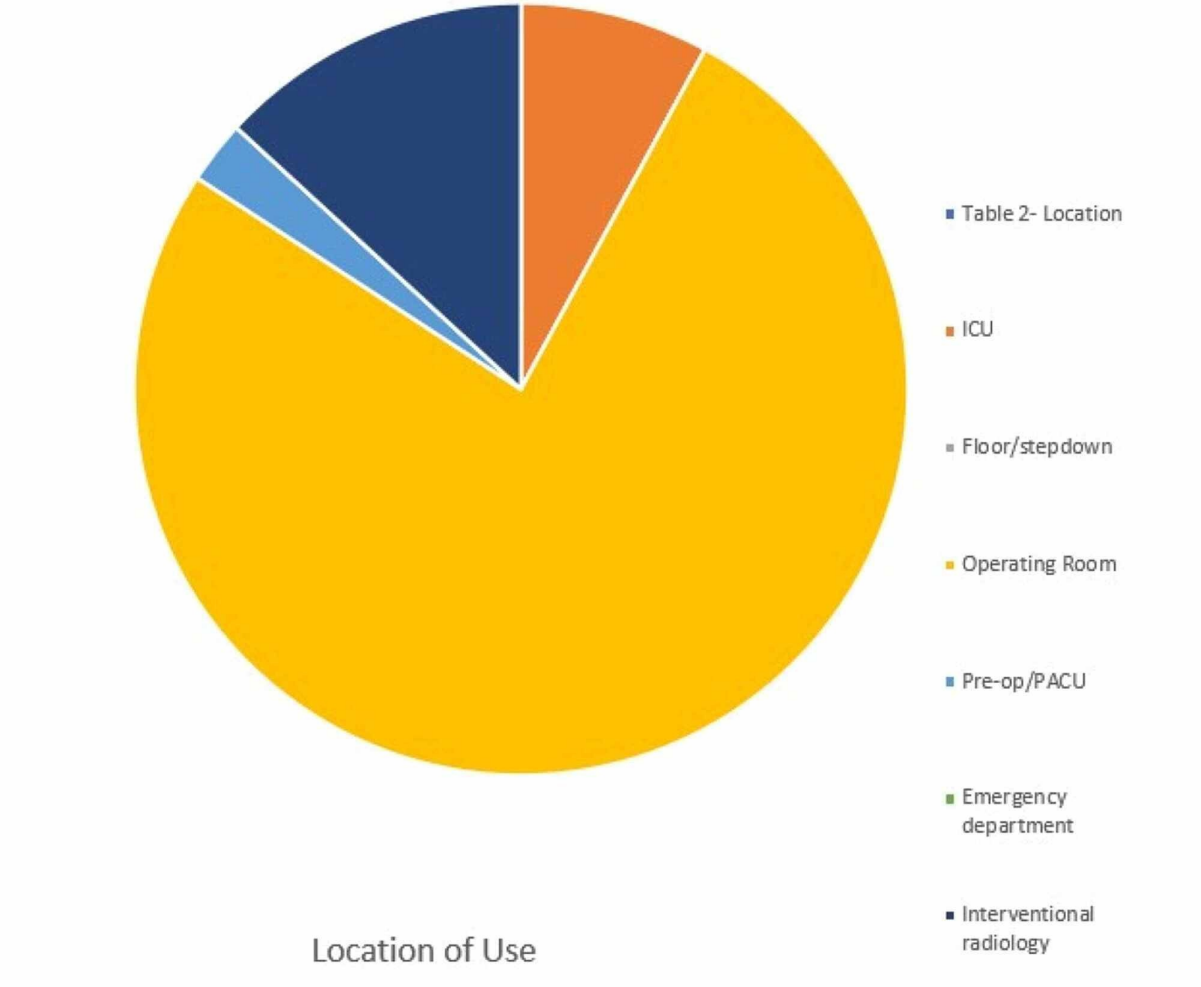
Location of intubation with Airway Box during procedure (intubation/extubation) PACU: post-anesthesia care unit

The surveys were reported from the following clinical areas:

29 were performed in the operating room, three in the critical care units, five in interventional radiology suites, and two in the perioperative setting (Figure 2).

Providers found the device to be readily available, with a score of 4.51/5, and a majority of providers, 60%, found the device easy to use, rating it either a 4 or 5 out of 5. However, the device was only utilized two times on a patient that was noted to have a difficult airway by the provider in the REDCap survey. Providers acquired a mean Mallampati score of 1.75, and 1.40 mean laryngoscopic grade view.

**Figure 3:**
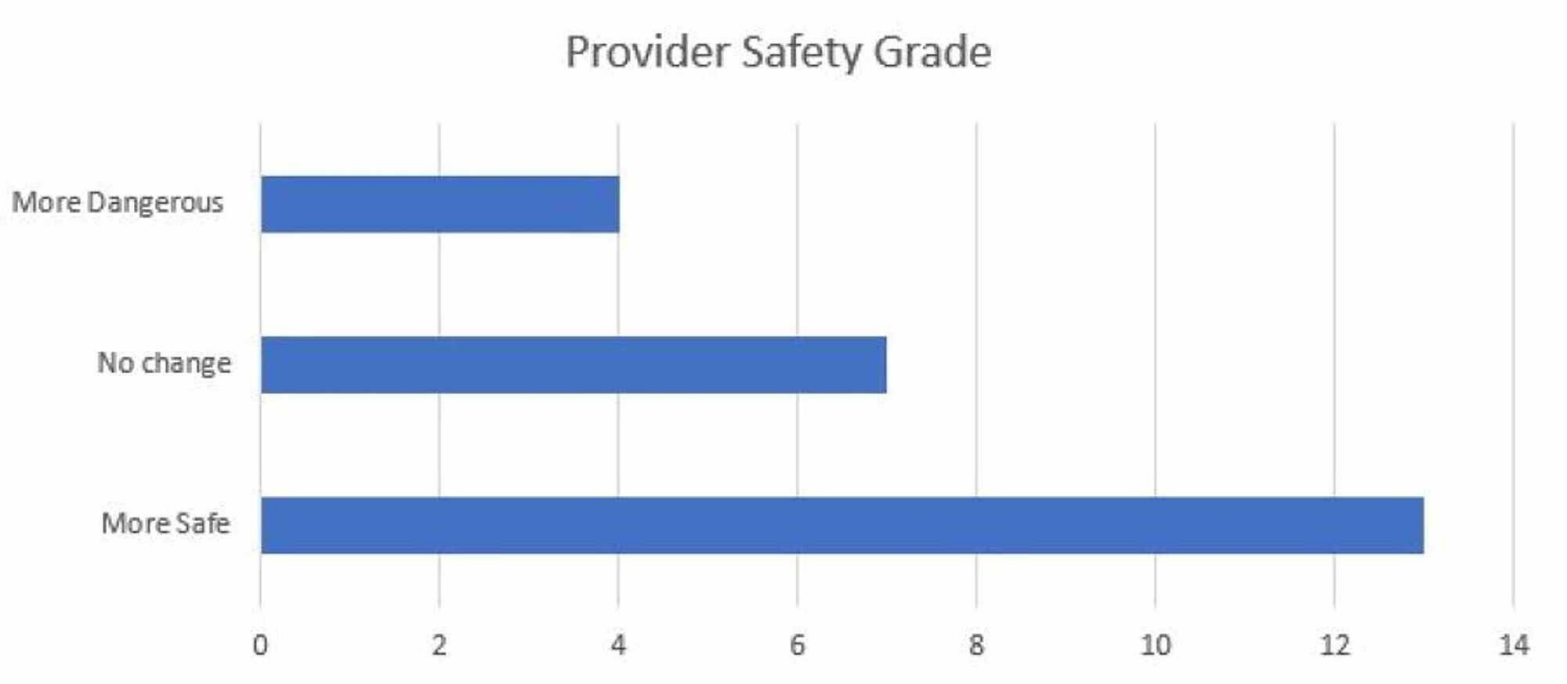
Provider safety designations for device implementation and overall procedure safety

**Figure 4:**
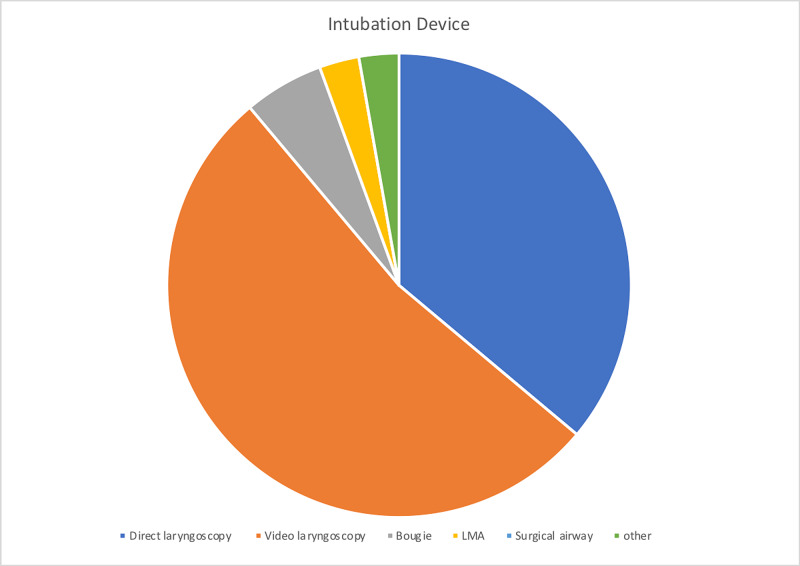
Devices utilized with airway box during procedure (intubation/extubation) LMA: laryngeal mask airway

**Figure 5:**
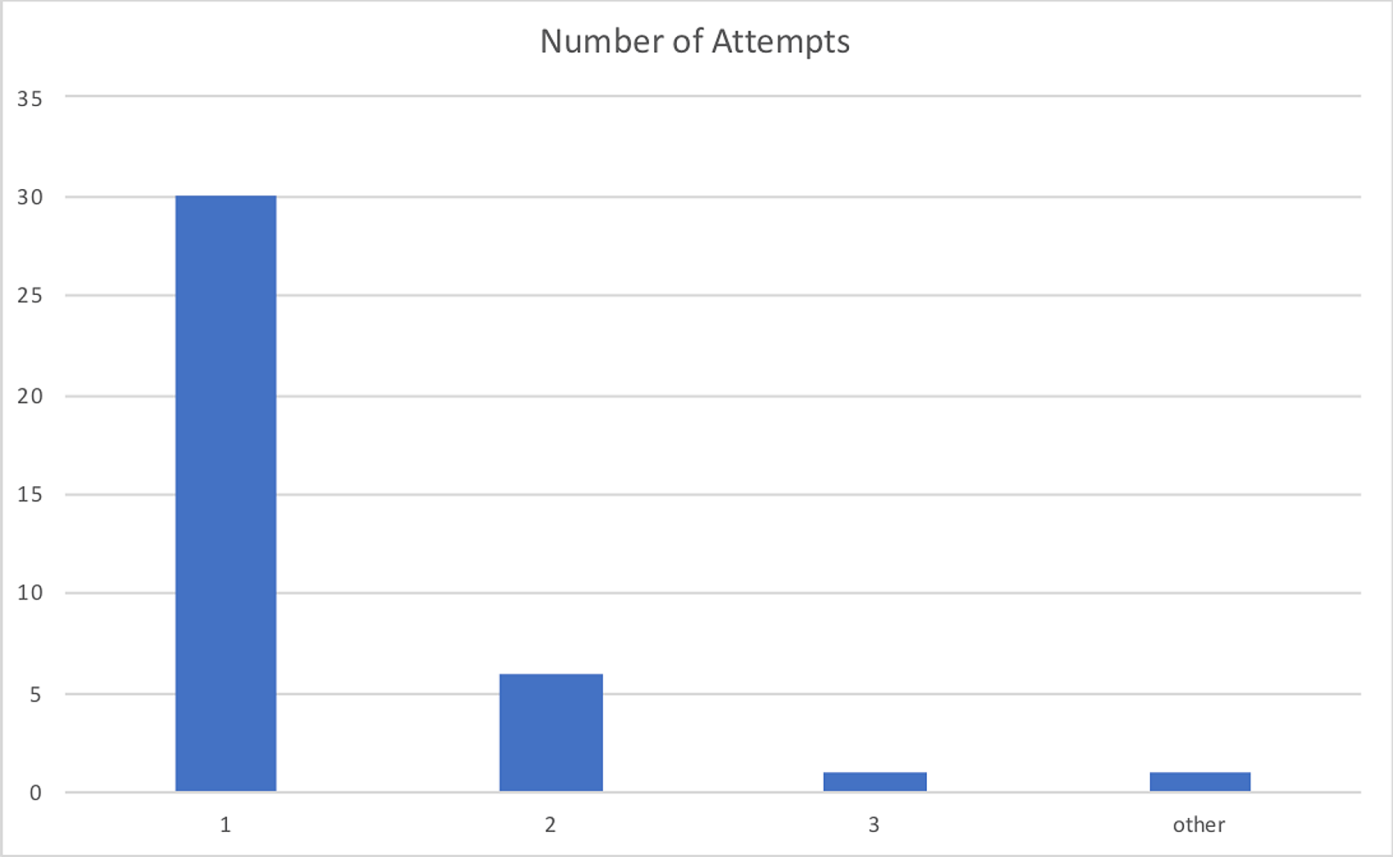
Number of attempts required by the provider with airway box during procedure (intubation/extubation)

**Figure 6:**
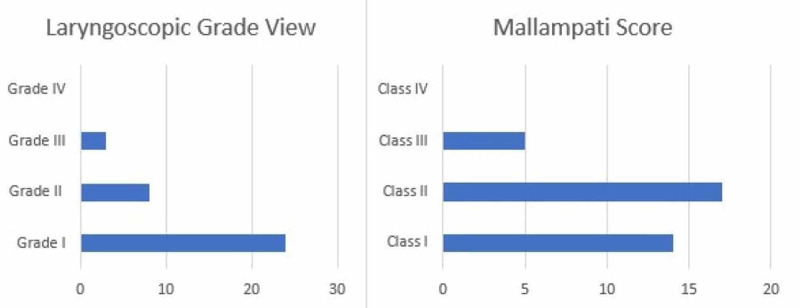
Laryngoscopic grade view and Mallampati score achieved with airway box during procedure (intubation/extubation)

The device was utilized primarily in the operating room (OR) accounting for 76% of surveys submitted. The box was used for intubation 82% of experimental entries. The device availability showed a mean rating of 4.51/5, 86% of providers rated the device as a 4 or 5 on a 1 to 5 scale. The device's ease of use was graded 3.51/5 on a 1-5 scale, 60% of providers graded the device a 4 or 5, and only 14% grading device with a 1/5. The average number of attempts is 1.29 with 78.9% successful intubations on the first attempt. The mean Mallampati score of 1.75 (14 class I, 17 class II, and five class III scores) and the mean laryngoscopy grade view was 1.40 (24 Grade I, eight grades II, and three grade II views) with 68% achieving a grade I view. In 94% of data entries, the provider deemed that the patient did not have a difficult airway, and the average BMI of patients was 29.42 with a maximum BMI of 45. Adjuvant protective methods were used in 36 of 39 attempts, including clear plastic drape, clear plastic drape with arm slits, and others. The devices used to perform showed that as expected either video or direct laryngoscope was selected in 88.89% of attempts. The average procedure time, from medication administration to intubation, was 3.26 minutes with a maximum time required of 11 minutes and a minimum of one minute required. Figures 3-6 illustrate the data.

## Discussion

The airway box is one of the several emerging solutions to possibly mitigate the risks of performing airway procedures during the SARS-CoV-2 pandemic. Due to the rapidly evolving nature of the pandemic, the intubation box has faced minimal clinical evaluation to ascertain whether the device can be effectively utilized by frontline workers. It is not only important to research the efficacy of the protection created by the device, but it is imperative to determine the impact on the provider’s ability to position the patient, obtain an adequate view of the glottis, and safely place (or remove) an airway in a timely fashion. Our study aimed to evaluate the utility of the device, identify limitations of the box, and to determine if there is a path to incorporation the box into routine use for airway procedures. This study indicates that the device was easy to use, readily available, and overall had a perceived safety benefit. However, it was still only used 38 total times during a period that saw 687 emergent intubations. Despite the benefits indicated in the survey, providers' apparent lack of interest was a significant limiting factor and could indicate future difficulty in expansion to PPE protocol. Stronger research on safety benefits would help education and commitment. 

Providers achieved an average Mallampti score of 1.75 and an average glottic view of 1.4 using the box with 78% being successful on the first attempt. This data paired with the direct survey question regarding ease of use (3.51/5) suggest that providers did not find significant difficulty in performing procedures with the device. However, the box was only used on a difficult airway twice and the mean BMI of study participants was 29.42. There has been conflicting data emerging regarding whether the device increases the difficulty of endotracheal intubation. A study conducted by Gould et al. found that the device does increase the procedural difficulty, citing the aerosol box limiting the provider's dexterity and space to work [10]. However, Wakabayashi et al.'s study found statistically significant prolongation of the procedure but concluded that the device's influence on procedural difficulty is clinically irrelevant [11]. Our study does not indicate that this device increases the difficulty of the procedure but does increase duration. The data and the controversy warrant further investigation into device use in more difficult airways. A study designed to incorporate more difficult airways could be implemented once it is determined if the potential decreased exposure risk warrants large-scale implementation. In a controlled setting, this would help identify possible unforeseen limitations created by the device.

The study’s subjective approach designed to ascertain the opinions from providers was an important step in the progression of the device but also limits the external validity of the study. The study does not attempt to evaluate particle concentrations and specific quantitative protective benefits. The box has been suggested to increase the time required to perform the intubation. In our study, the mean time required to successfully intubate the patient was 3.26 minutes. A simulation study conducted by Begley et al. found that without the airway box, no intubation took longer than one minute, but in our study, 58% took over one min and 17% took over two minutes [12]. Any increased time required to perform the procedure directly leads to an increased risk of hypoxia and negative outcomes. This could be addressed by training with the device and potential modifications to the design. When the airway box was utilized, participants reviewed the box positively, yet the box was only reviewed 38 times. This suggests the difficulty to acquire provider adherence regardless of their own perceived benefit to their personal safety. If further research supports the use of the device, the question may be asked if the device warrants a mandated protocol until sufficient adherence of use is acquired. National organizations could make recommendations supporting the use, but to gain support, several unanswered questions regarding the device would need to be investigated. Time is of the essence and it is becoming more apparent as this pandemic continues that the challenges recognized during the outbreak require innovative solutions.

Our data supports the emergence of the airway box as a possible addition to PPE and suggest further research and implementation. This study did not attempt to address possible modifications to the box that could address concerns established in other studies. One possible adjustment to device manufacturing could be the addition of suction and glove boxes to the device. The addition would not alter function, provider view, ease of operation but could limit any viral particles that would normally escape the device. This device will not eliminate the risk of exposure, but we hope it can significantly decrease risk in an effort towards creating a safer hospital with the stepwise implementation of sensible PPE, without impacting patient quality of care or expense.

Approximately 9% of SARS-CoV-2 patients require ICU admission, and of that group, 88% require intubation and mechanical ventilation [13] The airway box is, therefore, a leading contender to mitigate the risk during the procedure. The implications of this device could result in its addition to the SARS-CoV-2 PPE, with provider use adherence and sufficient device availability. Feedback from healthcare providers in our study supports the efficacy of the device to decrease direct exposure. 54% of the providers felt the device increased safety from exposure while only 16% found the device to decrease safety.

Our study design did not attempt to evaluate the rooms’ aerosolization particle concentration; however, we have considered the research from Melbourne, Australia suggesting the potential total viral load increases [14]. This study’s conclusion does not reference that the box is not pressurized and should not affect the overall aerosolization created during the event of aspiration or coughing. Specifically, they find that the aerosolization boxes may increase airborne particles through the holes in the box. The study uses saline nebulizers in large quantities which creates a high-volume pressurized spray that may not be representative of the standard execution of intubation or extubation. The device has two 6.5 cm access ports that do not create a pressurized spray and is designed to prevent direct spray of particles to the healthcare provider. Particle concentration in the air is significantly affected by air circulation, humidity, and atmospheric pressure within the room. The air within an operating room circulates at an average rate of 18-26 cycles per hour, which helps alleviate some of the risks that may result from using the airway box. While we do not intend to contradict or understate the validity of this study, it is important to consider risk-reward for the provider performing intubation. The room circulation paired with the standard PPE helps to decrease the impact of air particles within the room. Implementation of “aerosol clearance time” can help with room contamination. The study referenced above by Canelli et al. illustrating the particle distribution during the procedure and noted the aerosolization particle spread to a two-meter radius without the use of the device and with the device contained the spray within the box [4]. In this study, we have concluded that the airway box could serve as a productive barrier enclosure mechanism to limit potential spread during intubation but insist further research and modifications like the addition of suction in the box should be conducted to evaluate the efficacy.

## Conclusions

In conclusion, based on our study, we do recommend the use of the airway box as one of the solutions to limit transmission COVID viral particles during an aerosol generating procedure. Modifications and improvements such as the addition of suction, glove holes and clear drapes may provide added benefit to frontline clinicians. Despite receiving positive feedback, there was still limited utilization, further implementation would encounter significant resistance to change until it becomes standard of practice. 

We recognize many institutions and clinicians may be slow to adapt from currently employed standards of practice. Due to the magnitude and ramifications of this pandemic it may be prudent to possibly include mandated use of this device until its practice is accepted as normal operating procedure. 
